# Neurofibromatosis type 1 with huge intrathoracic meningoceles misdiagnosed as pleural effusion: A case report and literature review

**DOI:** 10.1186/s13019-024-02819-3

**Published:** 2024-05-30

**Authors:** Ningyi Chen, Wenjuan Li, Lingfeng Min, Qian Huang, Jiarong Bian

**Affiliations:** Department of Respiratory Medicine, Northern Jiangsu People’s Hospital Affiliated to Yangzhou University, Yangzhou, 225001 People’s Republic of China

**Keywords:** Intrathoracic meningocele, Neurofibromatosis 1, Thoracoscope, Spinal deformity, Differentiated diagnosis

## Abstract

**Background:**

Neurofibromatosis type 1 is a genetic disease that affects multiple organs and systems, leading to various clinical manifestations. In Neurofibromatosis type 1, rare intrathoracic meningoceles often occur alongside bone dysplasia. These meningoceles contain cerebrospinal fluid and can be mistakenly diagnosed as ‘pleural effusion’.

**Case presentation:**

In this case report, we mistakenly identified ‘cerebrospinal fluid’ as ‘pleural effusion’ and proceeded with drainage. This error posed significant risks to the patient and holds valuable implications for the future diagnosis and treatment of similar patients.

**Conclusions:**

In patients with Neurofibromatosis type 1 complicated by spinal deformity, there is a high incidence of intrathoracic meningoceles. Treatment strategies may differ based on the specific features of the lesions, and collaboration among multiple disciplines can significantly improve patient outcomes.

## Background

Intrathoracic meningocele is a rare condition characterized by the bulging of the meninges through the intervertebral foramen or a defect in the vertebral body. It is most associated with neurofibromatosis type 1 (NF-1), accounting for 60 to 85% of cases [1]. NF-1 is an uncommon chromosomal inherited disease with an incidence of approximately 1/2500-1/3000 in newborns [2]. These patients often develop scoliosis and meningocele in adulthood as a result of sclerotin dysplasia [3]. Initially, patients with intrathoracic meningocele may not exhibit any signs or symptoms. However, as the disease progresses, some patients may experience chest tightness or breathlessness due to compression of the lungs. Unfortunately, due to the rarity of the condition and a lack of expertise, the meningocele may be misdiagnosed as pleural effusion. As a result, patients may undergo unnecessary drainage of a mass pleural effusion, putting them at risk of complications and even death. In this article, we present a case of a patient with NF-1 combined with huge intrathoracic meningocele with NF-1 who was misdiagnosed as pleural effusion.

## Case presentation

A 43-year-old female patient with a history of NF-1 presented to the local hospital on 16 February 2023, complaining of chest congestion and dyspnea that had been ongoing for a month. The patient had a history of skin conditions, such as brown macule, since the age of 4. At the age of 14, she underwent surgical treatment for a subcutaneous mass in her right leg, which was diagnosed as NF-1 through pathology. Although the patient had no prior family history of NF-1, her son also suffered from the condition. Over the past three years, she had experienced intermittent coughing but had not sought medical attention due to mobility problems and feelings of inferiority. One month ago, she started experiencing chest tightness and dyspnea at rest. She sought care at a local hospital, where a massive pleural effusion was discovered in the right hemithorax. To determine the cause, the local hospital performed closed chest drainage on the patient. However, only a small amount of transudate was drained, which was not consistent with the patient’s condition of massive pleural effusion. Subsequently, the patient was referred to our hospital. Upon admission, physical examination revealed decreased breath sounds in the right lung. The patient presented numerous skin nodules with pedicles and café-au-lait spots scattered across the trunk. Additionally, a large soft mass was noted in the lower lumbar region, and several masses were found on the right lower extremity, leading to difficulty in walking. No neurological signs or symptoms were observed during the examination.

**Fig. 1:**
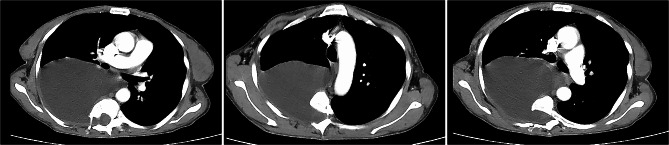
Chest CT scan revealed a large low-density mass in the right thoracic cavity that extended into the spinal canal. Scoliosis deformity in the thoracic spine, a defect on the right side of the T3-T5 thoracic vertebrae, and enlargement of the spinal canal and intervertebral foramen

**Fig. 2:**
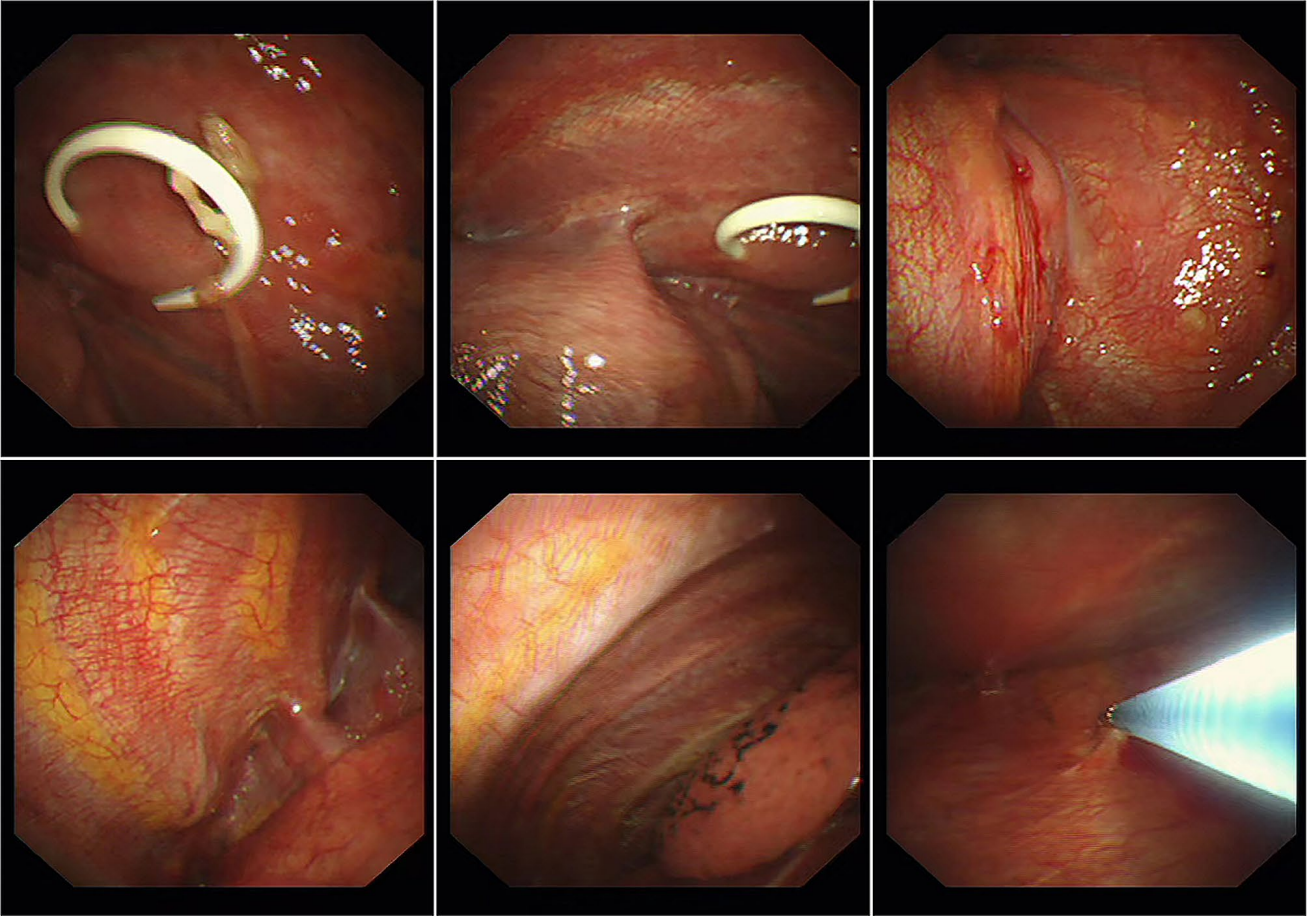
The thoracoscope confirmed the presence of a drainage tube within the thoracic cavity. The figures indicated minimal fluid accumulation and smooth pleurae, and a tissue biopsy was conducted

Chest computed tomography (CT) scan revealed a large low-density image in the right thoracic cavity, and a defect was observed in part of the vertebral plate in the thoracic spine (Fig. 1). The chest tube brought from local hospital was ineffective in draining the fluid, leading us to conduct a thoracoscopy to investigate the cause of the pleural effusion. The intraoperative exploration revealed that the drainage tube was in the thoracic cavity with only a small amount of fluid present. The pleurae appeared normal with no nodules, and the pleural biopsy results were normal as well (Fig. 2). Surprisingly, postoperative plain chest X-rays showed a significant pleural effusion still present in the right thorax, contradicting the findings of the thoracoscopy.

This raises the question of what caused the sudden accumulation of such a large amount of fluid in the pleural cavity and why it was not detected during the thoracoscopy. To address this issue, we replaced the original chest drainage tube with a new one guided by ultrasound. Following the insertion of the new tube, approximately 1000 ml of clear liquid was drained, leading to a significant relief in the patient’s chest tightness symptoms. However, she developed a severe headache the following day and found relief by lying flat on her back. Subsequent analysis of the hydrothorax revealed a white blood cell count of 11 × 10^6^/L, lactate dehydrogenase level of 36U/L, total protein level of 3.7 g/L, and glucose level of 3.21 mmol/L. Cytology examination confirmed the absence of malignant tumor cells in the pleural effusion. Furthermore, thorough examination and data analysis ruled out heart failure, hepatic and renal dysfunction, hypothyroidism, and hypoproteinemia.

**Fig. 3:**
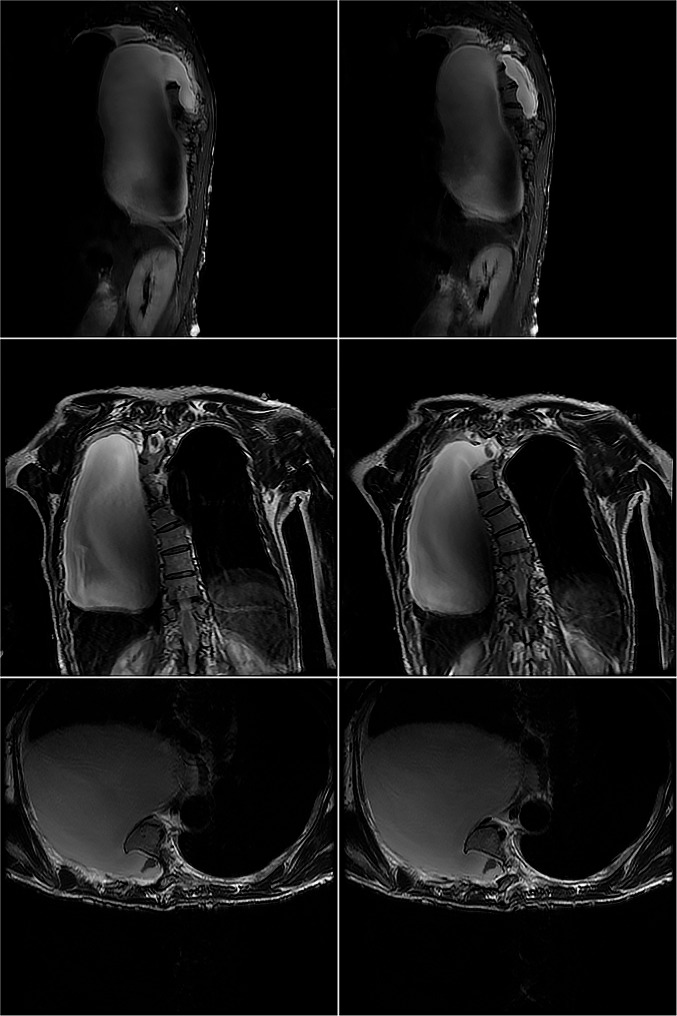
MRI revealed: 1) discontinuity between the T4-T8 vertebrae in both sagittal and horizontal axial views, 2) protrusion of the thoracic vertebrae towards the right, 3) communication between the right pleural effusion and the spinal canal. Most importantly, the MRI revealed that the ‘pleural fluid’ had similar intensity to CSF, indicating the presence of an intrathoracic meningocele

The pleural fluid was clear and transparent, with the patient developing a postural headache following the pleural effusion drainage. Upon analysis, we discovered that the fluid was cerebrospinal fluid (CSF) in actual. While CSF hydrothorax is commonly associated with traumatic injury or spinal surgery, this patient did not have a history of such events in the spinal column or chest. We reviewed the chest CT and identified a low-density image connected to the canalis vertebralis (Fig. 1). Subsequently, a thoracic magnetic resonance imaging (MRI) was performed (Fig. 3). The MRI revealed: 1) discontinuity between the T4-T8 vertebrae in both sagittal and horizontal axial views, 2) protrusion of the thoracic vertebrae towards the right, and 3) communication between the right pleural effusion and the spinal canal. Most importantly, the MRI revealed that the ‘pleural fluid’ had similar intensity to CSF, indicating the presence of an intrathoracic meningocele. During thoracoscopy, it was observed that the drainage tube, initially placed at a local hospital, had successfully entered the thoracic cavity instead of the bulging meninges, resulting in only a minimal amount of fluid being drained. Regrettably, our ‘successful’ fluid drainage procedure led to the patient experiencing postural headaches.

Upon recognizing the misdiagnosis, we promptly removed the drainage tube, disinfected the puncture site, and applied a sterile dressing. We invited neurosurgery, spine surgery, and thoracic surgery in a multidisciplinary discussion to consider surgical intervention. After comprehensive evaluation, it was determined that surgical procedures require collaboration across multiple disciplines, posing significant challenges and risks. Therefore, conservative treatment was advised for the time being, with a suggestion for patient to seek further care at a specialized hospital. After receiving conservative treatment, including anti-infection measures, the patient’s puncture site healed naturally, leading to a complication-free discharge without issues like pneumocephalus or meningitis. We conducted a follow-up call 3 months after the patient was discharged from the hospital. The patient sought surgical treatment at a more specialized hospital, where they were informed of the high risk and cost associated with the procedure. Ultimately, the patient opted for continued observation and follow-up, and is currently alive.

## Discussion

NF-1 is a rare genetic disease that affects multiple systems, including the skin, bones, joints, respiratory system, and nervous system [4]. Intrathoracic meningocele, although relatively uncommon, is often associated with NF-1. It has been observed that 60–85% of thoracic meningoceles occur in individuals with NF-1 due to dural hypoplasia and intervertebral foramen enlargement, which are the primary contributing factors to the development of intrathoracic meningocele. Intrathoracic meningocele can be asymptomatic, but when the bulging area compresses the lungs, it can cause symptoms such as chest tightness and dyspnea. Asymptomatic patients can be treated conservatively, while patients with obvious symptoms may require surgical intervention [5]. Surgery is the primary method for fully addressing intrathoracic meningocele in individuals with NF-1, necessitating collaboration across multiple disciplines. The specific surgical approach is determined by factors such as the size of the meningocele, extent of spinal deformity, and overall medical conditions [6].

To enhance our understanding of recent surgical methods, we conducted a comprehensive literature review. Using the PubMed database, we searched for articles with the keywords ‘(Meningocele) AND (Neurofibromatosis)’ from 2000 to 2024, resulting in a total of 90 relevant articles. The literature review based on the titles and abstracts, those not relevant to the topic of intra-abdominal meningocele, non-surgical treatment, or lacking specific surgical details were excluded. Ultimately, 21 articles on surgical treatment for intrathoracic meningocele related to NF-1 were obtained. Among the articles, 20 were case reports and 1 was a case series. Literature review comprised 21 patients diagnosed with NF-1 who received surgical intervention for intrathoracic meningocele. The data gathered encompassed details such as patient age, gender, symptoms, presence or absence of spinal deformity, size of the meningocele and location, as well as any history of previous diseases. Additionally, information on spinal or meningocele surgery, surgical techniques employed, postoperative complications, and prognosis during follow-up was documented (Table 1).

**Table 1:** Results of a literature review of surgically treated NF-1 patients with intrathoracic meningocele. CP: cystoperitoneal, VP: ventriculoperitoneal, LP: lumboperitoneal

Year	Age / Sex	Previous surgery	Symptom	Location / Meningocele size (cm)	Spinal malformation / Meningocele segment	Surgical approach	Postoperative complications		Treatment and follow-up
2024 [7]	51/F	-	Dyspnea	Single on the left / 13 × 14.6 × 15	Spinal deformity / T9	1. Thoracoscopic drainage 2. Meningeal resection and repair 3. Postoperative drainage	Headache while sitting	Symptoms disappeared	
2022 [8]	44/M	-	Paresthesia of lower limbs	Single on the right / 4.5 × 5.0 × 4.8	No spinal deformity / T3-T4	1. Vertebral resection 2. Meningeal resection and repair	-	Died of pneumonia 3 months later	
2021 [6]	56/F	Twice CP	Dyspnea	Single on the left / 9.9 × 12.5 × 18.3	Spinal deformity / C7-T2	1. Thoracotomy and transection of T2-T3 ribs 2. Attempt to detach the meninges failed	CSF leak	Thoracoscopic meningectomy repair and pleurodesis	
2021 [9]	41/F	T2-L2 fusion for scoliosis	Dyspnea	Multiple on the left / 13 × 19	Postoperative spinal fusion / -	1. Thoracoscopic drainage 2. Thoracotomy and meningectomy repair	Dyspnea relieved, but meningocele recurs	Surgery and no recurrence after 1 year	
2020 [10]	49/M	-	Dyspnea	Single on the left / Rupture size cannot measure	Severe spinal deformity / T3-T6	1. Thoracotomy and drainage 2. Meningeal repair.	-	Complete lung recruitment after two weeks	
2019 [11]	59/F	LP	Weakness in lower limbs	Single on the right / > 10	Thoracic spine left curvature / T1-T2	1. VP	-	Syringomyelia is reduced, but weakness of lower limbs is not relieved	
2017 [12]	43/F	Spinal fusion	Dyspnea, back pain	Single on the right / > 10	Thoracic spine right curvature / T8-T11	1. CP 2. Right posterolateral thoracotomy and meningectomy repair 3. Postoperative drainage	Meningocele recurred	Thoracotomy again, therapeutic pneumoperitoneum, postoperative drainage	
2017 [13]	50/F	-	Dyspnea, bilateral upper limb numbness	Single on the right / > 10	Spine right curvature Cobb 42° / T4-T5	1. Scoliosis correction 2. T4 and T5 total laminectomy, right transverse process resection, including resection of part of the proximal fourth and fifth ribs 3.Subcutaneous fat graft repair	-	Died of malignant tumor 30 months later	
2015 [14]	52/F	-	Dyspnea	Multiple on the right / 14 × 11.5 × 11 and 9.2 × 9.1 × 8.6	Spine right curvature / T4-5 and T5-6	1. CP 2. Thoracotomy and meningectomy repair 3. Postoperative drainage	Small amount of CSF leak	Meningocele subsided and the lungs expanded after 8 months	
2014 [15]	48/F	-	Dyspnea, chest pain	Single on the right / 21 × 11	Thoracic kyphosis / T4-T6	1. CP	Meningocele recurred after 9 months	Thoracoscopic meningocele plication	
2014 [5]	45/F	-	Chest tightness, chest pain	Single on the right / 15 × 15	Scoliosis / T2-T3	1.Thoracotomy and drainage 2. Meningeal resection and repair	-	The patient returned to normal after 2 weeks	
2014 [16]	43/F	-	Dyspnea, episodic syncope	Single on the right / > 10	Scoliosis / T11-T12	1.Thoracotomy and meningectomy repair 2. Spinal fusion 3. Pleurodesis	-	Symptoms disappeared 4 years after surgery	
2011 [17]	46/M	-	Dyspnea	Single on the right / 7.5	Scoliosis / T3-T4	1. Lumbar puncture and cerebrospinal fluid drainage 2. Thoracotomy and meningectomy repair 3. Postoperative drainage	-	The patient returned to normal after 1 year	
2011 [18]	48/M	-	Dyspnea, weakness in lower limbs	Single on the left / > 10	Kyphosis / T8	1. Thoracotomy, drainage, resection, and repair 2. Spinal cord decompression and fusion.	-	Symptoms disappeared	
2011 [19]	66/F	Peritoneal cavity shunt	Chest pain, hypovolemic shock	Single on the left / > 10	- / -	1. Thoracotomy to stop bleeding 2. Cerebrospinal fluid shunt replacement	Meningocele expanded after the hematoma shrank	Another thoracotomy was attempted to perform meningocele cerclage but failed	
2011 [20]	47/F	-	Dyspnea	Multiple both sides / > 10	Kyphosis / -	1. CP under local anesthesia	headache	Meningocele smaller after 24 months	
2008 [21]	60/F	-	Dyspnea	Single on the left / > 10	Thoracic kyphosis / T2-T4	1. CP under local anesthesia	-	Meningocele smaller after 2 months	
2003 [22]	59/M	-	Dyspnea, chest pain	Multiple on the left / 10–15	Thoracic kyphosis / -	1. Thoracotomy, drainage and meningeal resection and repair 2. Postoperative drainage	-	Returned to normal after 12 days	
2003 [23]	59/M	-	Dyspnea	Single on the left / > 10	Thoracic kyphosis / T2-T5	1. Posterior T2-T5 extensive laminectomy 2. Meningectomy repair	Lower limb weakness	Posterior C2-L2 long fusion	
2002 [24]	67/F	-	Dyspnea	Single on the right / 9	Scoliosis / T4-T5	1. Posterior T3-T5 extensive laminectomy 2. Meningectomy repair	Chest tightness	Surgery again for repair, and examination revealed no meningocele 4 months later	
2002 [25]	16/M	-	cough, chest pain	Multiple on the right / 5 and 8	Kyphosis / -	1.Thoracotomy and meningeal resection and repair	Paraplegia of lower limbs	Paralysis gradually recovered, and spinal fusion performed 6 months later	

The average age of the patients who underwent surgery was 49.9 years old, with 7 (33%) males and 14 (67%) females. The most common clinical symptom reported was dyspnea, with an incidence rate of 17/21 [5–7, 9, 10, 12–18, 20–24]. Followed by chest and back pain in 5/21, which was related to radiculopathy [5, 12, 15, 22, 25]. Spinal cord-related symptoms were observed in 4/21 patients, presenting as limb paresthesia, limb weakness, and ataxia [8, 11, 13, 18]. Additionally, hemorrhagic shock and syncope were each reported in one patient [16, 19].

Among the 21 patients, intrathoracic meningocele was observed on the left side in 9 cases and on the right side in 11 cases. Additionally, one patient presented with bilateral intrathoracic meningocele [20], while 5/21 patients had multiple intrathoracic meningoceles [9, 14, 20, 22, 25]. In 17/21 patients, the size of the intrathoracic meningeal cyst was greater than 10 cm, while in 4/21 patients, the size ranged between 5 and 10 cm [8, 17, 24, 25].

One patient had previously undergone cystoperitoneal (CP) shunt twice, but the meningocele recurred 6 years later. Following an unsuccessful attempt to transect the T2-T3 ribs and resect the meninges, the patient underwent thoracoscopic meningectomy repair and pleurodesis. Symptoms were completely resolved 5 months post-operation [6]. Another patient with a history of lumboperitoneal (LP) shunt developed symptoms of enlarged syringomyelia and lower limb weakness after surgery. This patient subsequently underwent ventriculoperitoneal (VP) shunt placement. While the syringomyelia reduced in size post-surgery, the lower limb weakness symptoms persist [11]. Five patients underwent CP shunt procedures for giant meningoceles measuring over 10 cm. Of these patients, 3 experienced a reduction in the size of their meningoceles within 1-year post-surgery [14, 20, 21], while 2 patients had recurring meningoceles that required additional surgical interventions, including thoracic surgery and thoracoscopic meningeal plication surgery [12, 15].

Among the 13 patients who underwent meningectomy repair for the bulge, 8 had thoracotomy [5, 9, 10, 16–18, 22, 25], 4 had meningectomy and repair following posterior laminectomy [8, 13, 23, 24], and 1 had meningectomy and repair through thoracoscopic surgery [7]. Out of the 8 patients with thoracotomy, only 1 experienced postoperative paraplegia complications, which resolved spontaneously after a few months [25], with the remaining patients showing a good prognosis. Of the 4 patients who had posterior laminectomy, 2 experienced postoperative walking difficulties and chest tightness, requiring further surgery [23, 24].

The primary objective of treatment is to alleviate lung compression and alleviate symptoms by resecting and repairing the meningocele. If clinical symptoms persist despite cerebrospinal fluid diversion, further surgical intervention may be necessary. Repairing bulging meninges can be achieved through either an internal or external approach. The internal approach may protect the nerve root, but performing a secure repair could be challenging due to the limited operating space. On the other hand, the external approach enables the resection or plication of the bulging meninges while completely ligating them. Whether it is thoracotomy, thoracoscopy, rib transection, or laminectomy, these methods all serve to expose the surgical field [6].

It is noted that many patients present with various spinal deformities, including scoliosis, kyphoscoliosis, costal deformities, and vertebral deformities such as spondylolisthesis, scalloping of vertebral borders, thinning of pedicles, enlargement of intervertebral foramen, and vertebral canal [26]. In our imaging analysis, 95% of the patients exhibited different levels of spinal deformity, with one patient having a history of spinal deformity correction surgery. The significance of spinal surgery in the treatment process should not be underestimated, especially for patients undergoing laminectomy or rib transection, as it may lead to spinal instability and require simultaneous spinal fusion. Hence, there is a strong emphasis on the importance of multidisciplinary collaboration.

Upon initial assessment of the patient in our study, we noted unexplained pleural effusion. Pleural effusion is a commonly observed condition in clinical practice, often associated with various underlying diseases. Despite advancements in diagnostic techniques, approximately 20% of pleural effusions still have an uncertain origin [26]. Thoracoscopy, a routine procedure, can be performed to identify the cause of unknown pleural effusions. Prior to performing thoracoscopy, it is important to thoroughly review chest CT images to identify any contraindications. For NF-1 patients with intrathoracic meningocele, the blind use of thoracoscopy can pose significant risks, including the development of meningitis, pneumocephalus, and even death [27].

CSF leakage into the chest cavity is a rare condition that usually occurs after trauma or surgery [28]. It is uncommon for patients to experience CSF leakage without a history of operation or trauma. β-2-Transferrin is a glycoprotein produced by neuraminidase in the brain and is predominantly found in CSF. It is present in only small amounts in cochlear perilymph, aqueous humor of the eye, and vitreous body. Laboratory techniques such as immunofixation, immunoblotting, gel electrophoresis, and isoelectric focusing are commonly used to determine β-2-transferrin content, all of which have high accuracy and specificity (over 90%) for CSF diagnosis. Moreover, studies have demonstrated that β-2-transferrin can be reliably detected in CSF samples stored at room temperature or refrigerated for 14 days [29]. Hence, β-2-transferrin serves as a reliable marker for determining CSF presence. In our case, although we strongly suspected the effusion to be CSF, we did not conduct a β-2-transferrin test on the extracted fluid. Instead, we selected MRI scanning due to its ability to clearly depict the relationship between the spinal canal, spinal cord, and nerve roots at the lesion site. Additionally, MRI can effectively differentiate fluids and distinguish between intrathoracic meningoceles, neurofibromas, and neuroblastomas. If the imaging reveals a correlation between pleural effusion and the spinal canal, along with spinal deformity and enlargement of the intervertebral foramen, intrathoracic meningocele should be taken into consideration [5].

## Conclusions

In patients with NF-1 complicated by spinal deformity, there is a high incidence of intrathoracic meningoceles. However, inexperienced doctors may resort to blind fluid drainage or thoracoscopy when managing these patients, resulting in complications such as postural headache, meningitis, pneumocephalus, and potentially fatal outcomes. Thorough history taking is essential when treating patients with these conditions. Treatment strategies may differ based on the specific features of the lesions, and collaboration among multiple disciplines can significantly improve patient outcomes.

## Data Availability

No datasets were generated or analysed during the current study.

## Abbreviations

NF-1: Neurofibromatosis Type 1
CSF: Cerebrospinal Fluid
CT: Computed Tomography
MRI: Magnetic Resonance Imagining
CP: Cystoperitoneal
VP: Ventriculoperitoneal
LP: Lumboperitoneal
